# Association between Twenty-Four-Hour Ambulatory Blood Pressure Variability and Cerebral Small Vessel Disease Burden in Acute Ischemic Stroke

**DOI:** 10.1155/2022/3769577

**Published:** 2022-10-18

**Authors:** Jun Shen, Lu Yang, Ziwei Xu, Wenshi Wei

**Affiliations:** ^1^Department of Neurology, Huadong Hospital of Fudan University, 221 West Yan An Road, Shanghai 200040, China; ^2^Clinical Research Center for Dementias and Cognitive Impairments, Huadong Hospital of Fudan University, 221 West Yan An Road, Shanghai 200040, China

## Abstract

**Objective:**

This study is aimed at investigating the association between the twenty-four-hour ambulatory blood pressure variability monitoring (ABPM) and cerebral small vessel disease (cSVD) burden in acute ischemic stroke (AIS) patients.

**Methods:**

115 AIS patients with demographics, vascular risk factors, 24 h ABPM, and brain magnetic resonance imaging (MRI) were retrospectively enrolled. 3.0 T MRI was used to assess cSVD burden by combining four MRI markers including white matter hyperintensities (WMHs), cerebral microbleeds (CMBs), perivascular spaces (PVS), and lacunes. Correlation analysis was conducted to detect whether ABPM was associated with cSVD burden in AIS patients.

**Results:**

115 AIS patients with mean age 68.77 ± 10.26 years and 75.7% male were enrolled in this study. 112 AIS patients (97.4%) had at least one cSVD marker. Spearman correlation analysis indicated that hypertension was positively correlated with cSVD burden (*ρ* = 0.21, *P* = 0.07). High-density lipoprotein (HDL) was negatively correlated with cSVD burden (*ρ* = −0.21, *P* = 0.02). Blood pressure variability such as 24 h mean SBP (*ρ* = 0.23, *P* = 0.01), day mean SBP (*ρ* = 0.23, *P* = 0.01), and night mean SBP (*ρ* = 0.20, *P* = 0.04) was positively correlated with higher cSVD burden. Ordinal logistic regression analysis demonstrated that higher 24 h SBP SD and day mean SBP were independent risk factors for cSVD after controlling for other confounders.

**Conclusions:**

Higher BPV was significantly related to total cSVD burden in AIS patients. 24 h SBP SD and day mean SBP were independent risk factors for cSVD burden in AIS patients but not DBP or DBP variability.

## 1. Introduction

Cerebral small vessel disease (cSVD) is a broad category of cerebrovascular diseases with the pathological damages of small perforating vessels, capillaries, and venules in brain [1, 2]. The established markers of cSVD identified on MRI include white matter hyperintensities (WMHs), lacunes, cerebral microbleeds (CMBs), and enlarged perivascular spaces (EPVS) in the basal ganglia usually contribute to 20-30% cases of all ischemic strokes [3–5]. Considering the simultaneous occurrence of the MRI markers, the cSVD burden was used to comprehensively assess the total damage of cSVD by combining the four MRI markers [6, 7].

High blood pressure is recognized as a major and modifiable risk factor for cSVD [8]. Emerging evidence indicated elevated blood pressure was positively correlated with the risk of individual marker of cSVD [9]. However, the blood pressure fluctuated under normal psychological stress or physical activity, and blood pressure variability (BPV) over a period of time could truly reflect the status of blood pressure and efficiently predict the cardiovascular events [10]. The relationship between BPV and cSVD burden was reported in physical examination population and memory clinical population [11, 12]. Whereas, the association between BPV and cSVD burden in acute ischemic stroke is less well-recognized. Therefore, this study is aimed at investigating the association between BPV and cSVD burden in acute ischemic stroke patients.

## 2. Methods

### 2.1. Study Participants

Participants with acute ischemic stroke were retrospectively recruited from January 1, 2019, to January 1, 2020, in the Department of Neurology, Huadong Hospital of Fudan University. The participants met the following entry criteria: cerebral MRI scan, diffusion weighted imaging conferred new stroke occurrence, small vessel occlusion based on the TOAST classification, and 24 h ABPM. The exclusion criteria were as follows: patients with severe stroke and previous cerebral diseases, infection of the nervous system, head trauma, demyelinating diseases, neurogenerative diseases, and brain tumor.

The basic demographic information and vascular risk factors were also recorded: age, sex, history of hypertension, diabetes mellitus, glycosylated hemoglobin (HbA1c), smoking, homocysteine (Hcy), total cholesterol, triglyceride, high-density lipoprotein (HDL), low-density lipoprotein (LDL), and fasting glucose.

### 2.2. Twenty-Four-Hour ABPM Recording

24-hour ambulatory blood pressure was performed for all the participants enrolled in this study with the Spacelabs 90217A device (Issaquah, WA). The blood pressure monitor is conducted from 8 : 00 a.m. to 8 : 00 a.m. the next day. The monitor recorded participant's blood pressure every 30 min during the daytime (6 : 00-22 : 00) and every 60 min during the night time (22 : 00-next 6 : 00). Participants were informed to maintain normal physical activity as usual, whereas vigorous exercise was not allowed in the meantime. The effective rate of blood pressure data ≥70% is considered as valid. The metrics of ABPM were calculated by Spacelabs analysis software. 24 h mean systolic blood pressure (SBP) and diastolic blood pressure (DBP), daytime mean SBP and mean DBP, and night time mean SBP and DBP were included in this study. Standard deviation (SD) of SBP and DBP was considered as blood pressure variability.

### 2.3. MRI Acquisition and Assessments

All participants enrolled in this study underwent 3.0-T MRI scan within 7 days of admission in department of radiology, Huadong Hospital. MRI sequences used in this study were listed as follows: *T*1-weighted imaging, *T*2-weighted imaging, fluid-attenuated inversion recovery imaging (FLAIR), susceptibility-weighted imaging (SWI), and diffusion-weighted imaging (DWI). MRI scan parameters were set for the sequences as followings: *T*1-weighted images (repetition time (TR) = 2,530 ms; echo time (TE) = 3.43 ms; inversion time = 1,100 ms; field of view (FOV) = 256 mm × 256 mm; voxel size = 1 mm × 1 mm × 1 mm; flip angle = 8°; 144 sagittal slices); *T*2-weighted images (TR = 6,000 ms; TE = 125 ms; FOV = 230 mm × 230 mm; flip angle = 90°; slice thickness = 5 mm, gap = 1 mm; 80 axial slices); FLAIR images (TR = 8,500 ms; TE = 81 ms; FOV = 230 mm × 230 mm; flip angle = 150°; slice thickness = 5 mm, gap = 1 mm; 80 axial slices); SWI (TR = 27 ms; TE = 20 ms; FOV = 208 mm × 230 mm, flip angle: 15°; slice thickness = 1.5 mm; 80 axial slices); and DWI (TR = 2501 ms; TE = 98 ms; acquisition matrix = 128 × 192; FOV = 230 mm × 230 mm; flip angle = 90°; slices = 18; section thickness = 6 mm; intersection gap = 1.0 mm; *b* values = 0 and 1000 s/mm^2^).

We rated the total MRI cSVD burden according to an established ordinal scale. cSVD burden was appraised by two neurologists who were blinded to participants' information. The presence of each lacunes, WMH, CMBs, and EPVS were counted as 1 point. The total cSVD burden ranged from 0 to 4 points.

### 2.4. Statistical Analysis

Continuous variables were reported as means (standard deviations), and categorical variables were presented as frequencies (percentages). Trend tests were performed to detect whether there are significant upward/downward trends in the level or prevalence of variables of interest, from group CSVD burden 0, through 1, 2, and 3, to 4. To achieve this purpose, linear regression model treating group CSVD burden as continuous variable was used for continuous variables, and Cochran-Armitage test was used for categorical variables. Spearman correlation analysis was used to assess the correlation of vascular risk factors with cSVD burden. Stepwise ordinal logistic regression was performed to investigate the correlation between blood pressure variability and cSVD burden adjusting for age, hypertension, and HDL. All analyses were conducted with JMP pro 11.0 (SAS Institute Inc., Cary, N.C., USA), and *P* < 0.05 was considered statistically significant.

## 3. Results

### 3.1. Characteristics of the Participants

The demographic, clinical, and ABPM characteristics of the participants in this study were listed in Table 1. We enrolled 182 acute ischemic patients in the Neurology Department of Huadong Hospital from August 1, 2019, to October 31, 2020. Twenty-nine patients without valid ABPM data were excluded, and thirty-eight patients with severe stroke were excluded.

The participants were divided into five groups according to cSVD burden, 3 (2.6%) participants had no cSVD marker, 21 (18.3%) participants had one cSVD marker, 33 (28.7%) participants had two cSVD markers, 34 participants had three cSVD markers, and 24 (20.9%) patients had four cSVD markers. Trend tests were performed to detect whether there were significant upward/downward trends in the level or prevalence of variables of interest, from group CSVD burden 0, through 1, 2, and 3, to 4. The variables of age, hypertension, and HDL were statistically different among the five groups. Sex, diabetes, smoking, total cholesterol, triglyceride, LDL, fasting glucose, HbA1C, and Hcy did not differ significantly among the patients with different cSVD burden.

### 3.2. Association between Vascular Risk Factors and cSVD Burden

In Table 2, Spearman correlation analysis was used to analyze the correlations between interested variables screened from trend tests in Table 1 and cSVD burden in AIS patients. Although there was a trend that the elder of the participants, the seriousness of the cSVD burden. The correlation coefficient between aging and cSVD was not significantly obvious (*ρ* = 0.17, *P* = 0.07). Hypertension was positively correlated with cSVD burden (*ρ* = 0.21, *P* = 0.07). With the cSVD burden increased, HDL level was decreased significantly (*ρ* = −0.21, *P* = 0.02). Spearman correlation analysis indicated that blood pressure variability such as 24 h mean SBP (*ρ* = 0.23, *P* = 0.01), day mean SBP (*ρ* = 0.23, *P* = 0.01), and night mean SBP (*ρ* = 0.20, *P* = 0.04) were positively correlated with higher cSVD burden.

### 3.3. The Association of cSVD Burden with Blood Pressure Variability by Ordinal Logistic Regression

Table 3 presents the results from the multivariable ordinal logistic regression models. In model 1, when the other confounders were not adjusted, the association between the listed variables (age, hypertension, SBP, BPV, and HDL) and cSVD burden remained significant. However, ordinal logistic regression analysis demonstrated that aging, hypertension, higher 24 h SBP SD, and day mean SBP were independent risk factors for cSVD after controlling for other confounders. The negative association between HDL and cSVD burden remained significant even after adjustment for age, sex, smoking, hypertension, diabetes, HbA1C, Hcy, and BPV.

## 4. Discussion

Our main findings of this retrospective study demonstrated that increased BPV was associated with total cSVD burden in patients diagnosed with acute ischemic stroke in our hospital. 24 h SBP SD and day mean SBP levels increased with the MRI burden of cSVD after adjustment for other risk factors. Age is an independent risk factor for cSVD burden, whereas HDL is a protective factor on cSVD burden in acute ischemic stroke.

Mounting evidence investigated the association between BPV and individual cSVD markers. Elevated BPV was reported to be correlated with WMH severity and brain volume reduction in healthy participants [13]. A large cohort study indicated that higher SBP variability was associated with cognitive decline via total WMH volume instead of deep or periventricular WMH volumes [14]. Higher SBP variability correlated with deep and infratentorial CMBs progression, and elevated DBP variability was positively related with development of new CMBs in deep regions; however, they did not find the association between BPV and WMH progression [15]. Higher SBP levels were independent risk factor for EPVS in basal ganglia after controlling for blood pressure in the participants for physical examination [16]. These studies suggest that BPV was correlated with individual cSVD markers; however, cSVD markers usually coexist in one participant especially the patients suffering from acute ischemic stroke. It is necessary to comprehensively observe the association between cSVD burden and BPV.

The association between BPV and cSVD burden is well known in specific human populations, but these results are not consistent. Our study displayed the correlation between 24 h SBP SD, day mean SBP levels, and cSVD burden which were in agreement with previous research, but not all. Yang et al. found that even after controlling for confounders, SBP levels and 24 h SBP variability were correlated with cSVD burden in participants from physical examination department [11]. Rianne et al. provided the clinical evidence that day-to-day systolic BPV was an independent risk factors for cSVD burden except diastolic BPV or mean BP in participants from memory clinic [12]. Fan et al. demonstrated that greater SBP wSD and DBP wSD of patients with cerebrovascular diseases related to the total cSVD burden progressed [17].

Age and vascular risk factors such as hypertension, smoking, homocysteine, LDL, and diabetes contributed to the risk of cSVD [18, 19]. Our results also showed that age and hypertension are to be associated with a higher risk of cSVD burden; moreover, we found the protective role of HDL on cSVD burden. These findings agree with previous research that higher HDL levels were associated with lower WMH volume and increased risk of small vessel stroke [20]. In order to disentangle the association between BPV and cSVD burden, the risk factors such as age, hypertension, and HDL factors were controlled in model 2. Our results indicated that 24 h SBP SD and day mean SBP were independent risk factors for cSVD burden but not DBP or DBP variability.

Our study has some limitations. First, this study recruited participants suffering from acute ischemic stroke in hospital. The patients were selected from a single center which might exist selection bias. Second, it is a small sample retrospective study, and the association between BPV and cSVD burden cannot be well established comparing with large population based longitudinal study. Third, this study used 24 h ABPM to measure BPV. However, day-to-day blood pressure monitoring efficiently reflects the true BPV of patients rather than 24 h ABPM. Finally, the results in this study should be interpreted with caution because the blood pressure may not be authentic due to the acute ischemic stroke.

Future studies could focus on the association between BPV and cSVD burden among different times after acute ischemic stroke. Meanwhile, future multicenter prospective and longitudinal studies could better explain the effect of BPV on cSVD burden in acute ischemic stroke.

## 5. Conclusions

We have shown that 24 h SBP SD and day mean SBP were independent risk factors for cSVD burden in acute ischemic stroke patients but not DBP or DBP variability.

## Figures and Tables

**Table 1:** Characteristics of the study participants.

Variables	cSVD burden (*n* = 115)					*P* for trend
	0 (3, 2.6%)	1 (21, 18.3%)	2 (33, 28.7%)	3 (34, 29.6%)	4 (24, 20.9%)	
Age, years	58.3 ± 10.5	67.8 ± 10.2	67.6 ± 12.6	69.2 ± 8.1	71.9 ± 8.84	0.04
Female gender, %	1 (33.3)	5 (23.8)	10 (303)	10 (29.41)	3 (12.5)	0.34
Hypertension, %	2 (66.7)	12 (57.1)	27 (81.8)	26 (76.5)	22 (91.7)	0.02
Diabetes, %	0 (0)	8 (38.1)	13 (40.6)	14 (42.2)	9 (37.5)	0.60
Smoker, %	1 (50.0)	5 (23.8)	13 (39.4)	15 (44.1)	9 (37.5)	0.42
ABPM, mmHg						
24 h mean SBP	124 ± 7	140 ± 16	143 ± 16	146 ± 16	148 ± 14	0.01
24 h mean DBP	74 ± 3	79 ± 10	80 ± 12	78 ± 9	80 ± 9	0.75
24 h SBP SD	11 ± 2	19 ± 3	13 ± 4	15 ± 4	16 ± 4	0.02
24 h DBP SD	8 ± 2	11 ± 3	10 ± 2	10 ± 2	11 ± 3	0.28
Day mean SBP	123 ± 10	141 ± 17	144 ± 15	148 ± 17	150 ± 14	0.007
Day mean DBP	75 ± 3	79 ± 10	80 ± 11	80 ± 10	81 ± 9	0.47
Day SBP SD	10 ± 2	14 ± 3	12 ± 4	15 ± 5	16 ± 5	0.02
Day DBP SD	7 ± 1	10 ± 3	9 ± 3	10 ± 3	10 ± 3	0.28
Night mean SBP	122 ± 2	133 ± 16	141 ± 19	142 ± 17	140 ± 16	0.07
Night mean DBP	71 ± 6	77 ± 10	79 ± 14	76 ± 11	75 ± 10	0.76
Night SBP SD	10 ± 2	12 ± 5	12 ± 4	13 ± 4	13 ± 4	0.10
Night DBP SD	7 ± 1	10 ± 4	9 ± 3	10 ± 3	9 ± 4	0.94
Total cholesterol	4.65 ± 1.36	4.45 ± 0.98	4.52 ± 1.50	4.32 ± 1.49	4.34 ± 1.44	0.59
Triglyceride	1.77 ± 1.13	1.2 ± 0.34	1.84 ± 2.40	1.31 ± 0.59	1.66 ± 1.12	0.78
HDL	1.64 ± 0.28	1.25 ± 0.22	1.25 ± 0.25	1.23 ± 0.25	1.14 ± 0.25	0.01
LDL	2.60 ± 1.42	2.82 ± 0.79	2.48 ± 0.79	2.42 ± 0.92	2.39 ± 0.71	0.10
Fasting glucose	4.57 ± 0.87	6.64 ± 2.69	6.31 ± 2.42	6.39 ± 2.13	5.80 ± 1.69	0.59
HbA1C%	6.03 ± 0.76	7.87 ± 2.62	6.72 ± 1.85	7.04 ± 1.75	6.65 ± 1.19	0.23
HCY	12.23 ± 7.82	12.78 ± 4.70	16.29 ± 14.22	15.02 ± 9.08	15.6 ± 6.28	0.42

Abbreviations: cSVD: cerebral small vessel disease; ABPM: ambulatory blood pressure variability monitoring; DBP: diastolic blood pressure; SBP: systolic BP; SD: standard deviation; HDL: high-density lipoprotein; LDL: low-density lipoprotein; HbA1c: glycosylated hemoglobin; HCY: homocysteine.

**Table 2:** Correlations between vascular risk factors and cSVD burden.

Variables	Correlation coefficient	*P*
Age	0.17	0.07
Hypertension	0.21	0.02
24 h mean SBP	0.23	0.01
24 h SBP SD	0.24	0.01
Day mean SBP	0.23	0.01
Day SBP SD	0.23	0.01
Night mean SBP	0.20	0.04
Night SBP SD	0.20	0.03
HDL	-0.21	0.02

Abbreviations: cSVD: cerebral small vessel disease; SBP: systolic blood pressure; SD: standard deviation; HDL: high-density lipoprotein.

**Table 3:** The association of cSVD burden with blood pressure variability by ordinal logistic regression.

Variables	Model 1	Model 2
	Odds ratio (95% CI)	Odds ratio (95%) ∗
Age, year	1.03 (1.00, 1.07)	1.05 (1.02, 1.09)
Hypertension (0 = *n*, 1 = yes)	2.57 (1.15, 5.73)	3.25 (1.41, 7.53)
24 h mean SBP, mmHg	1.03 (1.01, 1.05)	/
24 h SBP SD, mmHg	1.10 (1.01, 1.20)	1.09 (1.00, 1.20)
Day mean SBP, mmHg	1.03 (1.01, 1.05)	1.03 (1.01, 1.05)
Day SBP SD, mmHg	1.10 (1.02, 1.19)	/
Night mean SBP, mmHg	1.02 (1.00, 1.04)	/
Night SBP SD, mmHg	1.07 (0.99, 1.16)	/
HDL, mmol/l	0.72 (0.48, 1.07)	0.21 (0.05, 0.84)

Abbreviations: cSVD: cerebral small vessel disease; SBP: systolic blood pressure; SD: standard deviation; HDL: high-density lipoprotein.

## Data Availability

The raw data supporting the conclusions of this article will be made available by the authors, without undue reservation.
